# Clavicular stress fracture in a cricket fast bowler: A case report

**DOI:** 10.1186/1752-1947-2-306

**Published:** 2008-09-19

**Authors:** Jeremy AF Read, Phillip Bell

**Affiliations:** 1 Royal Surrey County Hospital, Egerton Road, Guildford, Surrey, GU27XX, UK; 2 BUPA Wellness Centre, White Lyon Court, Barbican, London, EC2Y 8EY, UK

## Abstract

**Introduction:**

Whilst rare, stress fractures of the clavicle have been described in other sports. To our knowledge, this is the first reported case of a stress fracture of the clavicle occurring in a cricket fast bowler.

**Case presentation:**

A 23-year-old professional cricket fast bowler presented with activity related shoulder pain. Imaging demonstrated a stress fracture of the lateral third of the clavicle. This healed with rest and rehabilitation allowing a full return to professional sport.

**Conclusion:**

This injury is treated with activity modification and technique adaptation. In a professional sportsman, this needs to be recognised early so that return to play can be as quick as possible.

## Introduction

This case presents an international level cricketer who developed an activity related shoulder pain which, after investigation, was demonstrated to be a stress fracture of the lateral clavicle. Stress fractures are well recognised in athletes, particularly in weight bearing bones and are often related to changes in training regime or intensity. Stress fracture of the clavicle is, however, an unusual injury and has not been described in this sporting group.

## Case presentation

A 23-year-old professional right-handed fast bowler presented with a 4-month history of presumptive bowling related right shoulder and upper anterior chest pain that was eased by rest. A winter season of low intensity cricket and high intensity weight training in Australia, plus a bowling symposium in India was followed by twice daily 60-minute bowling practice sessions during which he started to experience pain. A rheumatologist found no abnormal clinical signs and a thoracic spine MRI was reported as normal.

Clinical examination (PB) revealed mildly protracted shoulders, with minor functional winging of the scapular and weakness of the supraspinatus. The pectoralis minor was also tight. Shoulder range of motion was full, cervical spine examination was normal as were subacromial impingement tests, acromioclavicular stress tests and superior labrum anterior and posterior (SLAP) provocation tests. The glenohumeral joint was stable to examination and there was bony tenderness over the lateral end of the clavicle.

**Figure 1:**
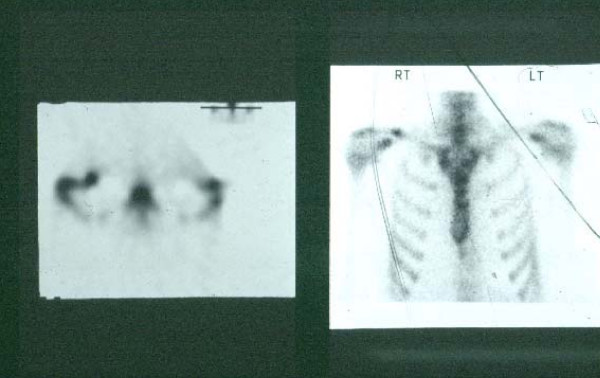
**Single photon emission computed tomography scan of shoulders**. A single photon emission computed tomography scan showing increased uptake in the lateral clavicle consistent with the site of pain.

**Figure 2:**
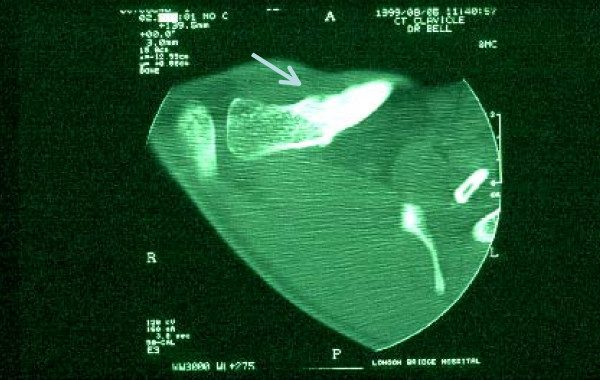
**Computed tomography scan of clavicle**. Computed tomography slice showing lucent line (arrowed) with adjacent sclerosis consistent with healing stress fracture.

A single photon emission computed tomography (SPECT) scan demonstrated a focus of increased uptake in the lateral third of the clavicle (Fig. 1) and a CT scan (Fig. 2) that revealed sclerosis and a lucent line with associated sclerosis confirmed a stress fracture.

He was rested from bowling and upper body weight training and rehabilitated with emphasis on scapular stability and rotator cuff strengthening until there was no residual local bony tenderness and he was asymptomatic with press-ups. He returned to professional cricket without recurrence.

## Discussion

Stress fractures are common in a sporting population, accounting for over 20% of all fractures in collegiate athletes [1]. In cricket, fast bowlers are at risk of stress fractures of the pars interarticularis [1,2] related to incorrect technique, overuse, and poor preparation, but, to our knowledge, stress fracture of the clavicle has never been reported in cricketers.

Stress fracture of the clavicle has been reported, following radical neck dissection [3], in a Catalan, human tower builder who had other team members standing on his shoulders [4], and a 'cable-maker' who spent his day lifting a heavy drum on his shoulder whilst tightening bolts several hundred times a day [5]. There are also reported cases in sports including a gymnast [6], a diver using an open hand water entry technique [7], a light weight sculler [8], a baseball 3rd base man [9], and a weight lifter [10], but none from cricket.

A stress fracture is a fatigue failure of bone and, as such, can result from repeated unusual, unopposed or uncoordinated loading. It can be proposed that the unopposed action of muscles acting at the lateral clavicle following radical neck dissection predisposed this patient to stress fracture. Repetitive direct loading of the bone in the cable maker and the tower builder as well as the repetitive torsional loading experienced by the diver indicate the possible aetiology in these cases. In the other cases, there are multiple factors that may have resulted in bone failure, for example, the intensity and nature of training and issues with technique and execution of their sport's specific activity.

There are several factors that may have contributed to the development of this injury in our patient, notably the intensity of net training and the increased level of upper body gym work that he was undertaking. There was muscle imbalance around his shoulder girdle that was addressed during rehabilitation, and though technical issues with his bowling were not directly considered, he underwent formal technical analysis on returning to his team.

It is possible to propose a mechanism for the development of this injury. It possibly relates to the activity of the anterior deltoid and pectoralis major on the inferior aspect of the clavicle, counteracted by the action of the trapezius and sternocleidomastoid acting isometrically. This is the mechanism hypothesised to have caused this injury in the rower. Alternatively, it could be the strut effect of the clavicle supporting the shoulder being heavily axially loaded during the overhead phase of the bowling action when it is almost vertical. This could then result in a bending force, leading to eventual failure.

## Conclusion

Clavicular stress fracture is a rare entity and it is not possible to determine the specific aetiology in this patient. However, recognition of the injury, rest and subsequent rehabilitation allowed him to return to top-level sport.

Clavicular stress fracture should be considered in the differential diagnosis of a cricket fast bowler presenting with bowling related shoulder and upper anterior chest pain in the dominant arm.

## Consent

Written informed consent was obtained from the patient for publication of this case report and accompanying images. A copy of the written consent is available for review by the Editor-in-Chief of this journal.

## Competing interests

The authors declare that they have no competing interests.

## Authors' contributions

PB was the treating clinician, while JR performed the background research and write up.
